# Study on Dihydromyricetin Improving Aflatoxin Induced Liver Injury Based on Network Pharmacology and Molecular Docking

**DOI:** 10.3390/toxics11090760

**Published:** 2023-09-07

**Authors:** Xiaoying Zhu, Silu Liu, Hongyan Pei, Weijia Chen, Ying Zong, Yan Zhao, Jianming Li, Rui Du, Zhongmei He

**Affiliations:** 1 College of Chinese Medicinal Materials, Jilin Agricultural University, Changchun 130118, China; m15886257839@163.com (X.Z.); liusilu0616@163.com (S.L.); zongying7699@126.com (Y.Z.); zhaoyan@jlau.edu.cn (Y.Z.);; 2 Jilin Provincial Engineering Research Center for Efficient Breeding and Product Development of Sika Deer, Jilin Agricultural University, Changchun 130118, China; 3 Key Laboratory of Animal Production and Product Quality and Safety, Ministry of Education, Jilin Agricultural University, Changchun 130118, China

**Keywords:** aflatoxin, dihydromyricetin, oxidative stress, network pharmacology, liver injury

## Abstract

Aflatoxin B1 (AFB1) is a toxic food/feed contaminant and the liver is its main target organ, thus it poses a great danger to organisms. Dihydromyricetin (DHM), a natural flavonoid compound, can be used as a food additive with high safety and has been shown to have strong hepatoprotective effects. In this experiment, PPI network and KEGG pathway analysis were constructed by network pharmacological analysis technique using software and platforms such as Swiss, String, and David and Cytoscape. We screened AFB1 and DHM cross-targets and pathways of action, followed by molecular docking based on the strength of binding affinity of genes to DHM. In addition, we exposed AFB1 (200 μg/kg) to mice to establish a liver injury model. Histological observation, biochemical assay, oxidative stress indicator assay, TUNEL staining and Western blot were used to evaluate the liver injury. Network pharmacological results were screened to obtain 25 cross-targets of action and 20 pathways of action. It was found that DHM may exert anti-hepatic injury effects by inhibiting the overexpression of Caspase-3 protein and increasing the expression of Bcl-2 protein. DHM (200 mg/kg) was found to reduce AFB1-induced liver indices such as alanine aminotransferase (ALT) and aspartate acyltransferase (AST), and attenuate hepatic histopathological damage through animal models. Importantly, DHM inhibited malondialdehyde (MDA) formation in liver tissue and attenuated AFB1-induced oxidative stress injury by increasing glutathione-S-transferase (GST) glutathione (GPX) catalase (CAT) and superoxide dismutase (SOD). Meanwhile, DHM also restored the expression of anti-apoptotic protein Bcl-2 and antioxidant proteins, Nrf2, Keap1 and its downstream HO-1, and down-regulated the expression of pro-apoptotic proteins Bax and Caspase-3 in AFB1-induced liver tissues. The results confirmed that liver injury caused by AFB1 exposure could be alleviated by DHM, providing valuable guidance for in-depth study of DHM in the treatment of liver-related diseases, and laying the foundation for in-depth development and utilization of DHM.

## 1. Introduction

**Figure 1:**
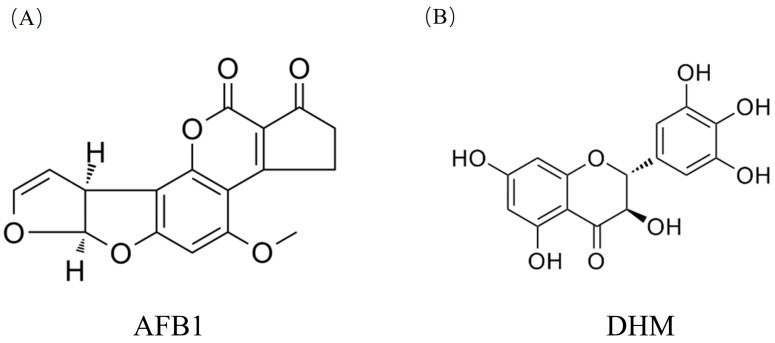
(**A**) Chemical structure formula of AFB1. (**B**) Chemical structure formula of DHM.

Aflatoxins (AFT) are toxic metabolites of *Aspergillus flavus* and *Aspergillus parasiticus* that are widespread in nature and frequently contaminate soil and crops, including peanuts [1] sorghum [2], barley [3] and, legumes and their by-products [4], as well as being considered a common food contaminant [5]. Among chemical hazards, AFT is one of the major food safety issues globally. In addition, several studies have confirmed that [6,7,8], chronic exposure to AFT poses a significant threat to human health globally. Based on data from the Rapid Alert System for Food and Feed (RASFF), it has been determined that AFT constitutes 20.9 percent of all releases reported between 2002 and 2019. In 2020, a total of 400 mycotoxin hazards were notified, of which AFT accounted for 92 percent [9]. AFB1(Figure 1A) is the most common type of AFT [10], it is also one of the most potent carcinogens found in AFT, causing significant contamination and damage to food and feed [11], and serious health and trade implications. If feed or food containing these toxins is consumed for a long period of time, it can cause serious liver damage to organisms and can even develop into liver cancer and other lesions [12]. In an Iranian study, 68.7 percent of 48 cereal samples collected were found to be contaminated with AFB1, with rice and wheat having the highest prevalence of AFB1 in infant cereal samples [13]. Abdolamir Allameh [14] who demonstrated that broilers fed only AFB1 showed balloon-like changes and severe necrosis of hepatocytes and even bile duct hyperplasia and death. 

The liver is the main target organ for AFB1. Qingqiang Xu [15] et al. demonstrated that the cell viability of human hepatocyte L02 decreased after exposure to AFB1, resulting in rapid oxidation and apoptosis, and that AFB1 down-regulated cellular antioxidant enzyme activity and exacerbated cell apoptosis. Fangju Liu [16] showed that exposure of ducks to AFB1 (60 μg/kg) significantly increased liver damage, cytochrome P450 (CYP450), and AFB1-DNA adducts, and induced apoptosis in hepatocytes. Currently, the problem of AFB1 contamination is still very serious and inevitable, so it is especially significant to find safer ingredients with low side effects to be protected from AFB1-induced liver injury (MILI) as an effective method in both clinical and animal husbandry settings. 

DHM (Figure 1B) is a natural flavonoid compound [17], also known as dihydromyricetin, and is the main active component in Ampelopsis Grossedentata [18]. It is traditionally used for clearing heat and removing toxins, calming the liver and lowering blood pressure, and unblocking the veins. Such active ingredients have fewer toxic effects and side effects than others, and according to the World Health Organisation, 80% of people rely on herbal medicines because of their significant use in our lives [19]. Interestingly, DHM showed a wide range of biological activities including antioxidant [20], anticancer [21], antitumour [22], antidiabetic [23] and neuroprotection [24], etc. Importantly, a study found that DHM has a significant therapeutic effect on liver injury and liver cancer, and that DHM promotes cell proliferation, inhibits apoptosis, and regulates intracellular redox balance [25]. Ping Qiu [26] et al. demonstrated that DHM could alleviate hepatic steatosis and ultimately reduce ethanol-induced liver injury by modulating the Keap-1/Nrf2 pathway. DHM is commonly used as a food additive, has a relatively high safety profile, and has many targets of action. Currently, DHM capsules have been sold in the United States as a nutraceutical to protect against alcohol-induced the liver damage caused by alcohol consumption [27]. This makes DHM a safe food ingredient, but whether DHM can alleviate the harm of AFB1 to liver has not been reported. 

In this experiment, we conducted a network pharmacological analysis to screen the cross-targets and action pathways of AFB1 and DHM. We then performed molecular docking. To evaluate the liver injury, we established a liver injury model and conducted histological observation, biochemical assay, oxidative stress indicator assay, TUNEL staining, and Western blot. The objective of this study is to determine whether DHM has a protective effect on liver injury caused by AFB1 and understand its possible mechanism of action. This study aims to provide a theoretical basis for the development of DHM in the prevention and treatment of this disease. 

## 2. Materials and Methods

AFB1 was purchased from Macklin.D ihydromyricetin was purchased from Shanghai yuanye Bio-Technology Co., Ltd. (Shanghai, China) BCA Protein Concentration Assay Kit, CAT Kit, MDA Kit, SOD Kit, ALT Kit, GPX Kit, GST Kit and AST Kit were purchased from Solarbio Biotechnology Co., Ltd. (Beijing, China). Electronic balance was purchased from Shanghai Jingtian Electronic Instrument Co., Ltd.(Shanghai, China).model FA2104A. The 1- -16K cryocentrifuge was purchased from Shanghai Trading Co., Ltd. (Shanghai, China). model D37520. H&E staining kit, Masson staining kit and TUNEL apoptosis assay kit. The 16K freezing centrifuge was purchased from Shanghai Trading Co., Ltd. (Shanghai, China). model D37520. H&E staining kit, Masson staining kit, and TUNEL apoptosis detection kit were purchased from Beyotime Biotechnology Co. (Shanghai, China). Rabbit anti-mouse, Nrf2, Keap1, Bax, Bcl-2, HO-1, Caspase-3, Cleaved-Caspase-3, and GAPDH monoclonal antibodies were purchased from Abcam (Cambridge, UK). All other chemical reagents used in the experiments were of analytical grade provided by Beijing Chemical Factory (Beijing, China).

### 2.1. Network Pharmacological Analysis of DHM and Potential Targets for Liver Disease

#### 2.1.1. Collection of Monomer Components and Targets Related to Aflatoxin-Induced Liver Damage

Firstly, the ingredient name dihydromyricetin was imported into the Pubchem database (https://pubchem.ncbi.nlm.nih.gov (accessed on 11 April 2023)) to obtain the ingredient SMILES (Simplified Molecular Input Line Entry System) and the chemical structural formula, and the obtained structural formula was imported into the structure. The obtained structural formula was imported into SwissTarget Prediction (http://www.swisstargetprediction.ch (accessed on 11 April 2023)), a similarity prediction target database, to predict the target. Then, we used GeneCards (https://www.genecards.org/ (accessed on April 11 2023)), OMIM (https://www.omim.org/ (accessed on 11 April 2023)), and the platform to obtain disease-related targets, and the disease name was “Aflatoxin induced liver injury” as the keyword to search for “Aflatoxin induced liver injury” related targets. The disease name was “Aflatoxin induced liver injury” as the keyword to search for “aflatoxin induced liver injury” related targets. We set the object as “human”, used the “VLOOKUP” function to match the target gene name, and then applied the software Venny (https://bioinfogp.cnb.csic.es/tools/venny/(accessed on 11 April 2023)) to obtain the target of the Chinese medicine. We used software to obtain the intersection target of the active compound and action target of the herbal medicine with the disease as a potential target of the composition for the treatment of aflatoxin-induced liver injury. 

#### 2.1.2. “Component-Target” Network Analysis

We prepared “network” and “type” files of compound genes, used Cytoscape 3.8.2 software, imported relevant files, and carried out topological analysis of the network, according to the Degree value (the number of connections of genes). According to the Degree value (the number of connections of genes), we adjusted the graphics, colour, transparency, and size of the target points, constructed the network diagram of “TCM components-targets”, and used Cytoscape software (version 3.8.2) to construct the network of “components-targets”. 

#### 2.1.3. Construction and Analysis of Protein-Protein Interaction (PPI) Networks

We entered the cross-targets into the String (https://string-db.org/(accessed on 12 April 2023)) platform and obtained their mutual inverse relationship, set the object as (homo sapiens), the interaction score required a maximum confidence level of 0.900, hid the free gene nodes, and used Cytoscape 3.8.2 software to visualise the PPI network. Network topology parameters were calculated using the Network Analyzer plug-in in Cytoscape 3.8.2 software. We selected “network Analyzer” to get the network topology parameters. Then, we imported the downloaded TSV file into Cytoscape software to make a PPI map. The intersecting targets were then imported into STRING website to obtain the PPI and TSV files of the protein interaction network, and then the target data of the TSV files were analysed by statistics and plotting in R language.

#### 2.1.4. GO and KEGG Enrichment Analysis

The bioinformatics open source software Bioconductor (http://www.bioconductor.org/ (accessed on 12 April 2023)) was used to install and run ClusterProfiler, Stringin, DOSE, and Pathview programs within R language to perform GO and KEGG function enrichment analysis of biological processes and visualize the results through the microbiology platform. The GO gene function annotates the role of target proteins in gene function in terms of biological Process (BP), cellular component (CC), and molecular function (MF) and is capable of discovering genes and gene products of all species. We discovered links between genes and gene product features in all species. Based on the KEGG website, enrichment analysis of the intersected targets and annotation of related signalling pathways was performed to elucidate the targets of drug therapy in signalling pathways. 

### 2.2. Molecular Docking

The top-ranked macromolecules and small molecules in terms of topological parameters were selected for molecular docking. Protein crystal structures were obtained from the RCSB PDB database (https://www.rcsb.org/ (accessed on 12 April 2023)) (PDB no.), or the Alphafold database (https://alphafold.ebi.ac.uk/ (accessed on 12 April 2023)) (beginning with AF). Docked small molecule libraries were obtained from the TCMSP database (https://old.tcmsp-e.com/tcmsp.php (accessed on 12 April 2023)) and built by searching for herbs. Protein crystal structures were dehydrogenated and hydrogenated using AutodockTools 1 and 2, and receptor structure preparation was performed. Preparations such as splitting of small molecule libraries were performed using Open Babel and Autodock programs. Docking was carried out using Autodock, and the final results were imported into Pymol software for saving AutoDock 4.2.6 software carried out hydrogenation and charge operations on compounds and proteins, performed molecular docking, saved the Binding Energy information, exported the docking file, and then carried out the visualisation and analysis. 

### 2.3. Animal Grouping and Drug Administration

Male BALB/c mice (5-week-old, body mass 20~22 g) were obtained from Changchun Yisi Experimental Animal Technology Co., Ltd. (Changchun, China) (SCXK-(JI)-2018-0023, China). The experimental protocols were carried out in strict accordance with the Guidelines for the Care and Use of Laboratory Animals approved by the Laboratory Animal Ethics Committee of Jilin Agricultural University. 

The mice were kept in a barrier environment with a barrier relative humidity of 30–50%, an ambient temperature of 18–26 °C, and alternating lighting and darkness for 12 h. All mice were free to take food or water, and completed 1 week of acclimatization, during which the mice were weighed weekly prior to gavage, the dosages of AFB1 and DHM were adjusted according to the changes in body weight, and the food intake of the mice was recorded. All mice were fasted and not hydrated after drug administration, weighed and executed, and the blood and liver were removed for liver index testing, liver index = (liver weight/body weight) × 100%. A small portion of the liver was sent in 4% paraformaldehyde for histological examination, and the rest of the tissues were frozen in liquid nitrogen and stored at −80 °C for further use. 

### 2.4. Analysis of Biochemical Indices

The serum markers of liver damage, glutamic alanine aminotransferase (ALT), and aspartate acyltransferase (AST) were examined using commercially available kits, following the methods specified in the catalogue, and the OD values were read at 505 nm. 

### 2.5. Detection of Oxidative Stress Indicators

Liver tissues were removed from −80 °C and MDA, GPX, GST, CAT, and SOD levels were assayed using commercial kits following the methods specified in the catalogue. Spectrophotometric measurements were determined using a BIO-TEK/mqx 200 r spectra ramax-M5 multifunctional microplate reader (Molecular Devices, Inc., Sunnyvale, CA, USA) at 450 nm. 

### 2.6. H&E and MASSON Colouring

Liver specimens were taken, fixed in 10% formalin, embedded in paraffin, sectioned, stained histologically with H&E and MASSON staining kits, and observed for histopathological changes in the liver under a light microscope. Then, a randomly selected slide area (200×) was evaluated for each sample and the mean score was calculated. 

### 2.7. TUNEL Fluorescent Staining

TUNEL fluorescent staining, according to the manufacturer, was used for the In Situ Cell Death Detection Kit (Roche Applied Science, Penzber, Germany). Liver sections were restained with hematoxylin before light microscopic analysis. 

### 2.8. Western Blot Assay

Western blot is an important method used to explore melecular mechanism of poisoning caused by toxic substances [28,29]. Proteins from the liver extracted from RIPA buffer Protein concentration of each group, were determined by BCA protein assay kit (Beyotime). Equal amounts of total protein (40 μg) were electrophoresed on sodium dodecyl sulphate- polyacrylamide gel electrophoresis (SDS-PAGE) and transferred onto polyvinylidene difluoride membranes (PVDF), which were sealed with 5% skimmed milk for 2 h. After three washes with TBS containing 0.1% Tween-20, the PVDF membranes were incubated with different primary antibodies. The PVDF membranes were incubated with different primary antibodies including Nrf2 (1:1000), Caspase-3 (1:1000), Keap1 (1:1000), HO-1 (1:1000), Cleaved- Caspase-3 (1:1000) Bcl-2 (1:1000), Bax (1:1000), and GAPDH (1:1000) for 12 h at 4 °C. The reaction was washed three times with TBST and then reacted with horseradish peroxidase-coupled secondary antibody. Protein bands were infiltrated using an electrochemiluminescence kit. Finally, the signal intensity of the protein bands was analysed by Image-Pro plus 6.0 software (Media). 

### 2.9. Statistical Analysis

Measurements were expressed as X ± s. All data were analysed by one-way ANOVA by SPSS 20.0 statistical software, GraphPad Prism 8 was used for plot collation and ImageJ was used for software for image analysis. 

All reference data were expressed as mean standard deviation and analysed statistically by SPSS 19.0 software (SPSS). Differences between experimental groups were analysed by one-way analysis of variance (ANOVA). *p* < 0.001, *p* < 0.001 or *p* < 0.05 were considered significant. 

## 3. Results

This section is divided by subheadings. It provides a concise and precise description of the experimental results, their interpretation, as well as the experimental conclusions that can be drawn.

### 3.1. Analysis of Potential Targets of DHM for the Treatment of Liver Injury

#### 3.1.1. Collection of Monomer Components and Targets Related to Aflatoxin-Induced Liver Injury

**Figure 2:**
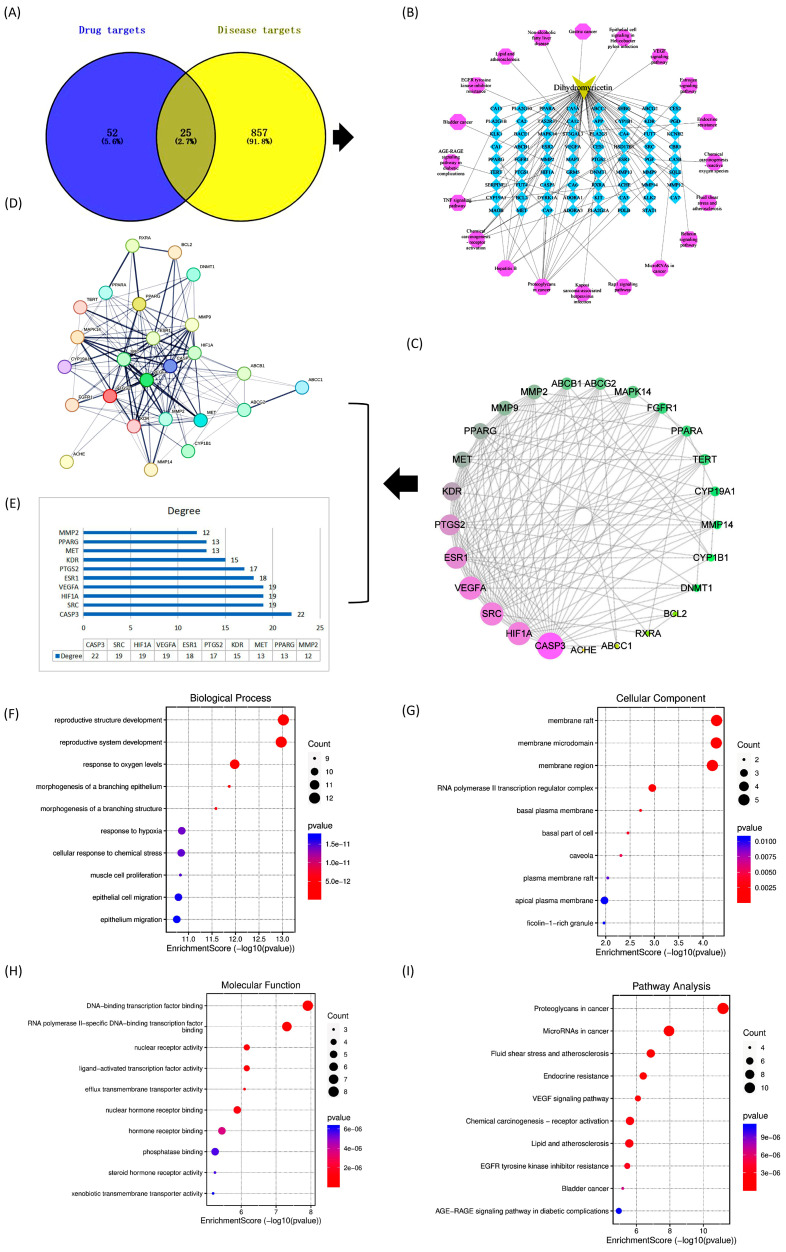
Network pharmacology-based prediction of multi-targets and pathways for the treatment of liver injury as well as functional annotations and enrichment pathways represented in the form of bubble diagrams. (**A**) Venn diagram of important compounds and their targets. (**B**) Network diagram. (**C**) Top 25 genes classified by degree method. (**D**) Expression of 25 target genes in human genes. (**E**) Bar graph of the top ten genes. (**F**) GO in biological processes (**G**) GO in cellular components (**H**) Molecular functions of GO (**I**) KEGG pathway analysis.

Fifty-two possible target genes were generated in the Stitch and Swiss Target Prediction databases for dihydromyricetin monomer components. After identifying promising targets for a compound, 857 genes associated with liver injury were collected from GeneCards (https://www.genecards.org/ (accessed on 12 April 2023)), and OMIM (https://www.omim.org/ (accessed on 12 April 2023)), databases. Later, Venn diagrams were used to predict the overlapping targets of dihydromyricetin and aflatoxin-induced liver injury. Finally, 25 anti-aflatoxin-induced liver injury genes found in dihydromyricetin were selected as the primary targets (Figure 2A). 

#### 3.1.2. Construction of Composite Target Networks

Dihydromyricetin corresponds to many different targets. In addition, 25 major targets and their associated pathways were used to construct a “component-target” network (Figure 2B) using Cytoscape software (version 3.8.2). This gave clear evidence that multiple targets may act synergistically when dihydromyricetin is used as an anti-aflatoxin-induced liver injury drug. 

#### 3.1.3. Construction and Analysis of Protein Interaction (PPI) Models

The 25 overlapping genes were then submitted to the STRING database for PPI network construction. The interactions between many targets throughout disease progression were characterized in the PPI network by nodes and their associated interactions (Figure 2C,D). Subsequently, using network analysis tools we found Casp3 (22), SRC (19), VEGFA (19),HIF1A (19), ESR1 (18), PTGS2 (17), KDR (15), MET (13), PPARG (13), and MMP2 (12), to be the highest (Figure 2E). The highest degree indicates that the target genes are highly connected to each other, which means that all of these genes could be potential targets. When comparing these results with those provided by functional annotation, four genes namely CASP3, SRC, HIF1A, and VEGFA were predicted as the major anti-hepatic injury targets and selected for molecular docking analysis. 

#### 3.1.4. GO and KEGG Pathway Enrichment

The biological functions of 25 shared targets were revealed based on GO enrichment results in biological processes, cellular components, and molecular functions (Figure 2F–H).The target genes were mainly involved in the development of reproductive structures, hypoxia response, cellular response to chemical stress, myocyte value-added and epithelial cell migration. 

The KEGG pathway results showed that the genes were significantly enriched in the cancer pathway, endocrine blockade, VEGF signalling pathway, lipid and atherosclerosis, and AGE-RAGE signalling pathway, etc. (Figure 2I).

### 3.2. Molecular Docking Analysis

**Figure 3:**
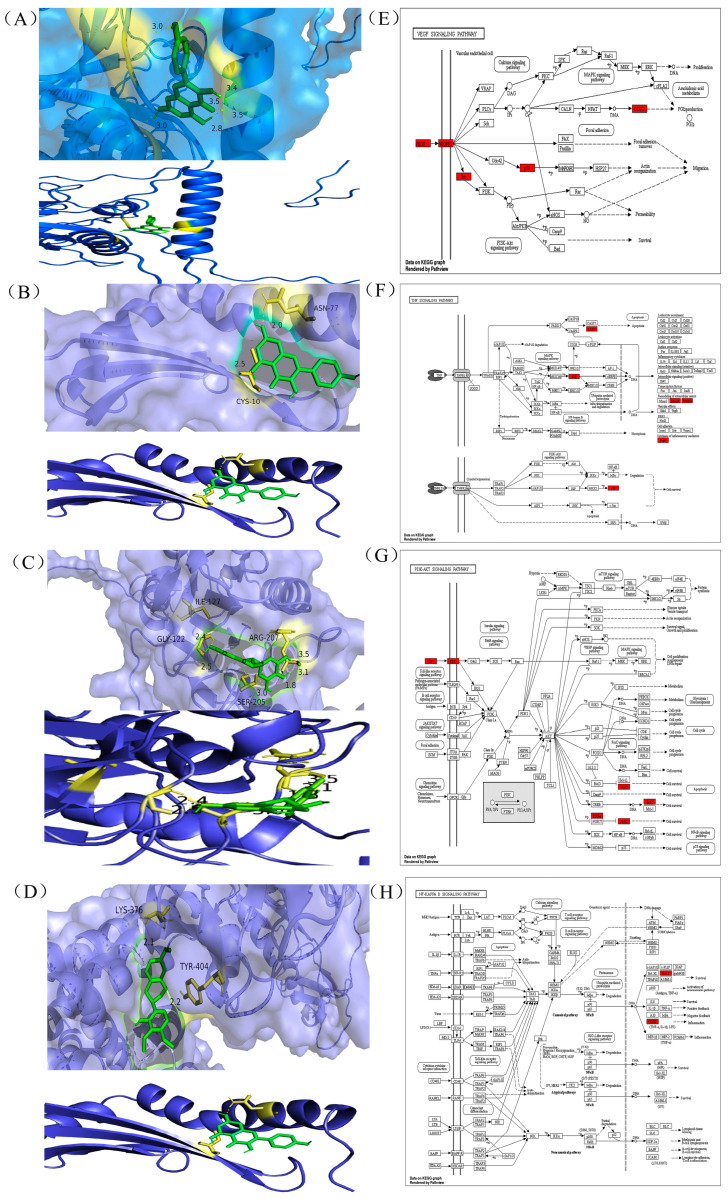
Binding affinity of genes to their compounds and prediction of which related signalling pathways (**A**) HIF1A (19). (**B**) SRC (19). (**C**) CASP3 (22). (**D**) VEGFA (19). Docking complexes are indicated where genes have a strong binding affinity to their compounds. (**E**) VEGF signalling pathway. (**F**)TNF signalling pathway. (**G**) P13K -AKT signalling pathway. (**H**) NF-κB signalling pathway.

Molecular docking methods are spatial and energetic recognition between molecules. Spatial matching is the basis for intermolecular interactions to occur, and energy matching is the basis for maintaining stable binding between molecules. The molecular docking studies show that target proteins such as CASP3, HIF1A, SRC, and VEGFA were able to bind stably to DHM compounds. The binding energy of DHM and its associated four target proteins (Table 1) was utilized using PyRx software and potential targets. The interactions were −5.03, −7.8, −6.4, and −5.86, kcal/mol, and the binding energies were less than −5 kcal/mol. Binding energy is used to assess the ability of a molecule to bind to a target. CASP3, HIF1A, SRC and VEGFA were visualized in a docking analysis to screen key active targets to minimize the risk of liver injury. The results showed that HIF1A had the highest binding affinity to DHM (Figure 3A) and interacted with HIF1A via van der Waals forces. SRC bound strongly to DHM (Figure 3B) and interacted with SRC via ASN-77 and CYS-10. CASP3 and VEGFA bound stably to DHM (Figure 3C,D), representing the strongest binding affinities between targets and compounds. DHM interacted with CASP3 via ILE-127, SER-205, GLY-122, and ARG-207, in addition, DHM interacted with VEGFA via LYS-376 and TYR-404. All significant targets binding dihydromyricetin exhibited van der Waals interactions, carbon-hydrogen bonding, Pi-anion, Pi-Pi stacking, and functioned through relevant signalling pathways (Figure 3E–H). In conclusion, the intermolecular interactions probed by molecular docking and the prediction of their binding modes and affinities can clearly demonstrate that this DHM plays an important role in the treatment of liver injury.

### 3.3. Effect of DHM on AFB1-Induced Liver Injury in Mice

**Figure 4:**
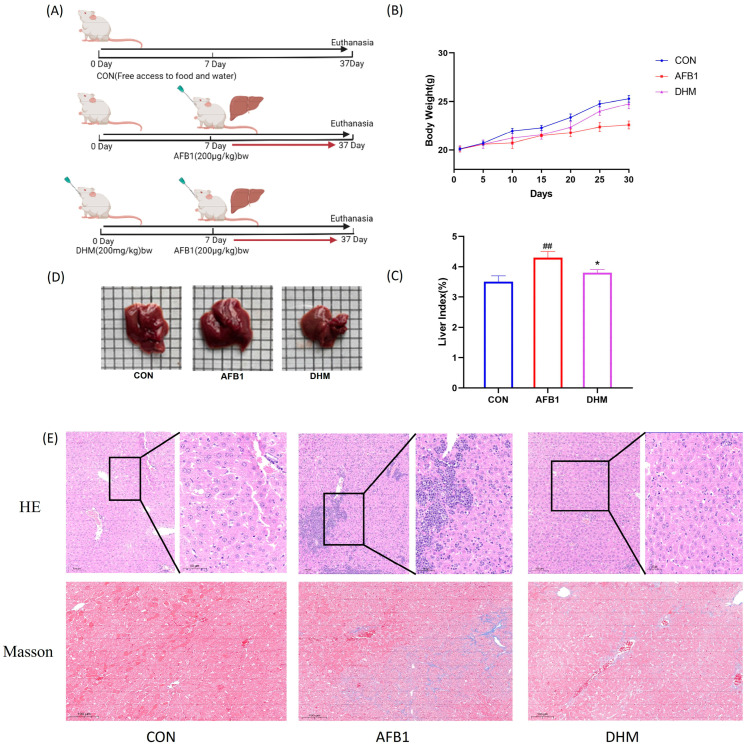
Protective effect of dihydromyricetin on AFB1-induced liver injury in mice. (**A**) Flow of drug administration in each group of mice (n = 8). (**B**) Body weight of mice in each group. (**C**) Effect of AFB1 on liver index in mice. (**D**) Liver injury in mice in each group. (n = 8). (**E**) Liver tissue H&E staining 200×, boxed magnification of H&E staining × 400, histological micrograph of a liver section stained by Masson. All data are expressed as mean ± standard deviation (n = 8). ^#^ represents statistically significant difference compared with the control group; * represents statistically significant difference compared with the AFB1 group. ^##^ *p* < 0.01, * *p* < 0.05.

After 1 week of acclimatization, the mice were numbered, and in the AFB1-exposed group, mice were exposed to 200 μg/kg of AFB1 by oral gavage per day (Refer to 24.787 μg/kg/d AFB1 daily average dietary exposure of Chinese high-consumption group). DMSO was used as a solvent for the carrier (with a final concentration of 2%), and the experiments lasted for 37 days, and the dose was fixed at 200 μg/kg (the daily intake equivalent to AFB1 is actually 3.5–4 μg). The dose of AFB1 was fixed at 200 μg/kg. (intake of AFB1 was actually about 3.5–4 μg),and of the DHM group was 200 mg/kg [30] which was ultrasonically mixed before use. Subsequently, 24 mice were equally divided into control, AFB1, and DHM_200_ (AFB1 + DHM) groups using the random number table method and the mice were pre-protected by gavage administration of DHM at 200 mg/kg. In addition, AFB1 gavage was carried out on day 7 (Figure 4A). Prior to modelling, the mice had glossy fur, normal diet and water intake, and a significant tendency to increase body weight. There was no significant difference in body weight between the groups. After a period of gavage of AFB1, there was a decrease in food intake, loss of coat lustre, and slow weight gain. However, when the mice in the DHM administration group gradually normalized their food intake, the body weight gradually returned to a steady growth (Figure 4B). 

Notably, compared with the control group, AFB1 significantly increased (*p* < 0.01) the liver index, which was significantly alleviated (*p* < 0.05) by the treatment of DHM (Figure 4C). Meanwhile, the macroscopic pictures of liver tissues showed that the liver tissues of mice in the AFB1 group were more hypertrophic compared with the control group, however, the liver tissues of the DHM group were roughly the same area as that of the control group (Figure 4D). Meanwhile, we used HE and Masson staining to observe the differences in liver structure in each group and to assess the effect of AFB1 on the liver. According to the histopathological results, the liver lobules of the control group had a clear structure with normal nuclei. AFB1 exposure caused severe liver damage, such as vacuolar degeneration, inflammatory cell infiltration, cell necrosis and swelling (Figure 4E), which was responsible for the effects of AFB1 on the liver, and this was the reason AFB1 led to the increase in the liver index. On the contrary, DHM treatment ameliorated the phenomenon of cellular vacuolization and inflammatory cell infiltration in necrotic and hepatic tissues (Figure 4E). 

### 3.4. DHM Alleviates AFB1 Exposure-Induced Liver Dysfunction and Ameliorates AFB1-Induced Oxidative Stress

**Figure 5:**
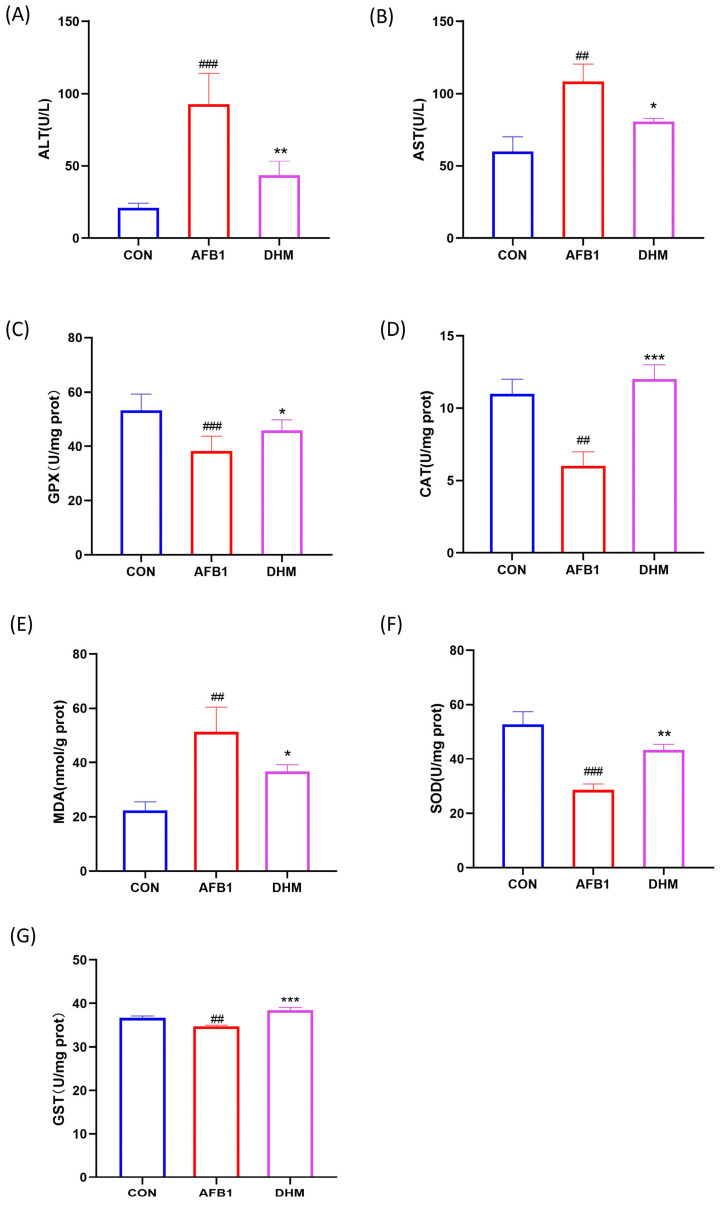
Effects of DHM on AFB1-induced liver function and amelioration of AFB1-induced oxidative stress. (**A**) Serum ALT activity of mice in each group. (**B**) Serum AST activity of mice in each group. (**C**) GPX activity in the liver of mice in each group. (**D**) CAT activity in the liver of each group of mice. (**E**) Concentration of MDA in the liver of each group of mice. (**F**) SOD activity in the liver of mice in each group. (**G**) GST activity in the liver of mice in each group. All data are expressed as mean ± standard deviation (n = 6). ^#^ represents statistical differences compared to the control group. * epresents statistical differences compared to the AFB1 group. ^##^ *p* < 0.01, ^###^ *p* < 0.001, * *p* < 0.05, ** *p* < 0.01, *** *p* < 0.001.

To investigate whether AFB1 exposure affected liver function in mice, we measured the serum activities of ALT and AST in mice. The results showed that the serum activities of ALT and AST were significantly increased in the AFB1 group of mice compared with the control group (*p* < 0.01), whereas a significant reversal of the activities of ALT and AST was observed after treatment with DHM (*p* < 0.05) (Figure 5A,B). It indicated that mice in the AFB1 group developed liver injury, and that treatment with DHM had a significant alleviating effect on AFB1-induced liver injury. 

Subsequently, the levels of MDA and the activities of antioxidant enzymes SOD, GPX, GST, and CAT in the livers of mice in the experimental group were examined. Compared with the control group, exposure to AFB1 decreased the activities of SOD, GPX, CAT and GST (*p* < 0.01) and increased the levels of MDA (*p* < 0.01), the above trend was significantly reversed by the administration of DHM (*p* < 0.01). Notably, AFB1 exposure appeared significantly impaired after DHM supplementation was significantly alleviated (Figure 5C–G). 

### 3.5. DHM Attenuates AFB1 Exposure-Induced Apoptosis in Liver Tissue

**Figure 6:**
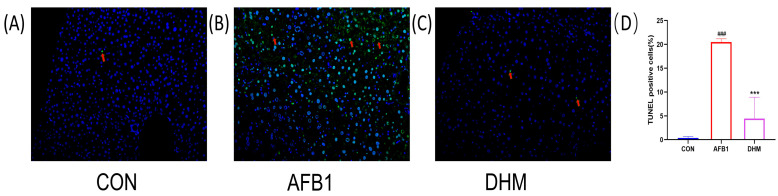
Effect of DHM on AFB1-induced apoptosis. TUNEL staining of (**A**) the control group, (**B**) the AFB1 group, and (**C**) the DHM group. Relative levels of fluorescence intensity were quantified (**D**). The presence of TUNEL-positive cells was assessed using an image analyser. And pointed out in detail with arrows. All data are expressed as mean ± standard deviation. ^#^ represents a statistically significant difference compared with the control group, * represents a statistically significant difference compared with the AFB1 group. ^###^ *p* < 0.001, *** *p* < 0.001.

When cells are in an apoptotic state, exposed 3’ - OH is labelled by the catalysis of terminal deoxynucleotidyl transferase and can thus be detected by fluorescence microscopy. Therefore, we assessed and quantified the extent of apoptosis in liver tissue using TUNEL staining. The results showed a significant increase (*p* < 0.001) in nuclear fragmentation and crumpling of cells in the AFB1 group compared with the control group (Figure 6B). Compared with the AFB1 group, the nucleus of the cells in the liver lobules were more rounded, the fluorescence intensity was more uniform, and the contours were more regular after DHM administration (Figure 6C). DHM treatment significantly reduced the number of TUNEL-positive cells induced by AFB1 (Figure 6D) (*p* < 0.001). The above data confirmed that DHM had a significant ameliorating effect on AFB1-induced apoptosis in liver tissue. 

### 3.6. DHM Attenuates AFB1-Induced Liver Injury by Ameliorating Oxidative Stress and Apoptosis

**Figure 7:**
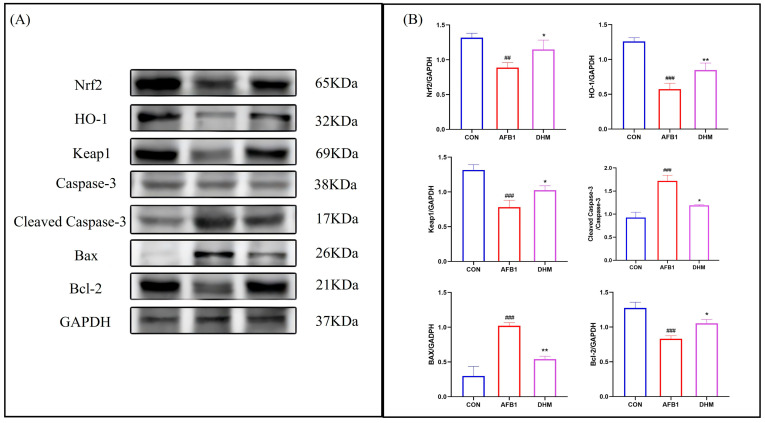
DHM can regulate the Nrf2/HO-1 signalling pathway and attenuate apoptosis to exert protective effects. (**A**) Protein expression of Nrf2, HO-1, Keap1, Cleaved-caspase-3, Caspase-3, Bax, and Bcl-2 was analysed using western blot analysis. (**B**) Relative protein expression was quantified by ImageJ analysis. All data are expressed as mean ± standard deviation (n = 3). ^#^ represents a statistically significant difference compared to the control group, * represents a statistically significant difference compared to the AFB1 group. ^##^ *p* < 0.01, ^###^ *p* < 0.001, * *p* < 0.05, ** *p* < 0.01.

To investigate whether DHM could attenuate AFB1-induced liver injury by ameliorating oxidative stress and apoptosis, we examined the expression of Nrf2, HO-1, Keap1, Caspase-3, Cleaved-caspase3, Bax, and Bcl-2 in liver tissues by Western blot (Figure 7A,B). 

The results showed that the expression of Keap1,Nrf2 and its downstream HO-1 was downregulated after AFB1 exposure compared to the control group, whereas DHM treatment resulted in up-regulation of Nrf2, Keap1, and their downstream HO-1 expression. In addition, AFB1 exposure was found to the upregulate the expression of the apoptotic proteins Bax and Cleaved-caspase3, while the expression of the anti-apoptotic protein Bcl-2 was downregulated when compared with the control group. However, DHM administration reversed these trends. The above results confirm that DHM exerts anti-apoptotic activity by regulating the Nrf2/HO-1 pathway to exert antioxidant activity to inhibit the expression of Bax and Cleaved-caspase3 proteins to promote the expression of Bcl-2 protein. 

## 4. Discussion

Our research focuses on investigating the protective effects of DHM against liver injury caused by exposure to AFB 1 virus. First of all, we searched for key bioactive compounds and effective targets from a large number of data through network pharmacology, which proved that DHM has multiple targets and pathways. We found that signal pathways such as tumour pathway, endocrine blocking, and VEGF signal pathway may be potential pathways to resist AFB 1-induced DHM liver damage. Subsequently, molecular docking using the binding energy of DHM and its related target proteins revealed that it mainly acted through CASP3, SRC, HIF1A, and VEGFA and their related signalling pathways. Overall, these results provide clear evidence that DHM plays an important role in the treatment of liver injury. Among them, the VEGF signaling pathway plays an irreplaceable role in the whole process of angiogenesis. Under pathological conditions, inflammation and the stimulation of tumour growth are the main factors for VEGF release.The VEGF signaling pathway is mainly composed of the key proteins VEGFR 2, Src, p 38 and COX 2. Up-regulation of VEGFR 2 protein can lead to liver injury by aggravating abnormal angiogenesis and capillarization of hepatic sinuses [31]. Inactivation of Src in mitochondria inhibits electron transfer and increases the release of reactive oxygen species, thereby maintaining JNK activation and promoting cell death and liver injury [32], SHC1 regulates cell proliferation and apoptosis, reactive oxygen species (ROS) production and oxidative stress [33]. P38γ can bind to Dlg1, a member of the membrane-associated guanylate kinase family, leading to liver injury, inflammation, and steatosis. This was significantly ameliorated when liver sections from p38γ knockout mice showed lower levels of oil-red O-stained dots and small leaky shapes [34]. It is well known that Caspase-3 is a basic apoptosis regulator [35], which can lead to cytoskeleton disruption, nuclear apoptosis, and other apoptosis-related cellular changes, suggesting that apoptosis may also be an effective way for DHM to treat AFB1-induced liver injury. In addition, several studies have confirmed that DHM can play a hepatoprotective role by its antioxidant effect. 

Subsequently, we verified the protective effect of DHM against AFB1-induced liver injury through oxidative stress and apoptosis. Histological observations are widely used as visual evidence of tissue damage caused by toxic substances [36,37]. AFB1 manifestations include swelling and necrosis of the liver, vacuolation, and severe inflammatory infiltration [38]. In addition, AFB1 significantly increased the expression of ALT and AST in liver [39]. In this study, after the liver injury model was established by gavage of 200 μ g/kg AFB 1, the mice showed a decrease in food intake and weight loss, and the levels of ALT and AST in the liver increased significantly, which could be improved by DHM treatment. Observation of HE staining results of histopathological sections further confirmed that DHM ameliorated the vacuolization, inflammatory cell infiltration, cell necrosis and swelling of liver tissues caused by AFB1 exposure, suggesting that DHM administration significantly ameliorated AFB1-induced liver injury. 

Oxidative stress is the process that occurs in AFB1-induced liver injury. Specifically, AFB1 first undergoes epoxidation at the 8-and 9-position of the terminal furan, which subsequently leads to the accumulation of reactive oxygen species (ROS) in liver tissue and the formation of oxidative stress adducts, which then ultimately leads to the deterioration of liver injury [40,41]. In order to resist oxidative stress and electrophilic damage induced by various poisons and carcinogens, cells have their own defence mechanisms. One of the most important defence mechanisms is Nrf2 induced production. Nrf2 is mainly located in the liver, kidney, lung, and other metabolic detoxification organs. Currently Nrf2 is considered a key transcription factor in regulating cellular resistance to xenobiotic substances and oxidative damage. Under normal circumstances, Nrf2-dependent transcription is inhibited by the negative regulatory factor Keap1. When cells are exposed to oxidative stress or chemo-preventive drugs, Nrf2 then dissociates from Keap1-mediated repression as a means of maintaining homeostasis of the cellular oxidation reduction response. When oxidative stress occurs Keap1 acts as a sensor of redox reaction, and the sensitive cysteine residue at its end, which is modified by the redox reaction to produce a highly efficient molecule. Then, this leads to a change in its conformation, which dissociates Nrf2 and trans-locates it into the nucleus of the cell, activating the expression of target genes and the transcriptional activity of antioxidant enzymes or drug transporters, thus exerting its role as an anti-oxidant against oxidative damage. Activation of the Keap1-Nrf2 signaling pathway initiates the expression of various downstream target proteins, which can regulate redox homeostasis and restore the body from oxidative stress to a normal physiological state. After activation, these target proteins can adjust the redox balance of the body and restore the body from the state of oxidative stress to a normal physiological state. The downstream target proteins regulated by Nrf2 have been divided into phase II metabolic enzymes, antioxidant proteins/enzymes, proteasomes/molecular chaperones, and anti-inflammatory factors. At present, quinone oxidoreductase-1 (NADPH)and heme oxygenase-1 (HO-1) are the most concerned and studied antioxidant proteins among many target proteins downstream of Nrf2. Glutathione peroxidase (GPX) is an important member of the antioxidant enzyme family which can effectively scavenge free radicals in living organisms by catalysing the reduction in hydroperoxides by glutathione (GSH), thus protecting cells from oxidative damage, and it has potential medicinal value for the prevention and treatment of many diseases caused by reactive oxygen species. Glutathione S-transferase (GST) is a group of enzymes related to the detoxification function of the liver. The enzyme is mainly found in the liver, and since the liver cytoplasm is rich in GST, the enzyme is rapidly released when the liver is damaged, leading to an increase in GST activity. At the same time, the AFB1-induced imbalance in the antioxidant defence system was accompanied by a decrease in CAT, SOD and GST content, as weii as a significant increase in MDA levels [42,43]. In addition, it has been proved that AFB1 can regulate the Nrf2/HO-1 signaling pathway, which is an antioxidant pathway leading to liver injury [44]. Shanji Liu [45], among others, found that the levels of inflammatory factors and apoptosis-promoting protein Caspase-3 increased significantly in mice with alcoholic liver injury, which was achieved by regulating the antioxidant pathway of Nrf2/HO-1. HIF-1 is an anoxic factor, and Titto Mathew showed that oxidative stress under anoxic conditions regulates the activation of different signalling pathways. Nrf2 is one of the key molecules which regulate the antioxidant responses of cells. Curcumin activates Nrf 2 under the conditions of hypoxia prevention, thereby triggering the up-regulation of antioxidants (GST, GPx, SOD, and GSH) and HO-1, thus maintaining the stable state of lung oxidation and antioxidation [46]. It is worth noting that, in this study, DHM treatment significantly inhibited the increase in MDA levels, but significantly increased CAT, SOD, GST, and GPX. Importantly, the Western blot results showed that compared with AFB1 group, the expression levels of Nrf2, HO-1 and Keap1 proteins in the control group and DHM group were higher, while the expression level of AFB1 decreased significantly after exposure. This shows that DHM can restore its antioxidant capacity by ameliorating oxidative stress damage.

Bax and Bcl-2 belong to the Bcl-2 gene family. Bcl-2 is an apoptosis inhibitor gene, and Bax not only antagonizes the apoptosis inhibitory effect of Bcl-2, but also has the function of promoting apoptosis; the subsequent activation of Caspase-3 ultimately leads to apoptosis [47]. The pro-apoptotic protein Bax and the anti-apoptotic proteins Bcl-2 and Caspase-3 regulate the apoptosis pathway. Overexpression of Bax and Bcl-2 apoptosis-related genes and pathological changes can lead to liver tissue damage [48,49]. AFB1 can lead to the up-regulation of pro-apoptotic factors Bax and Caspase-3, while the protein level of apoptosis inhibitor Bcl-2 decreases [50,51]. Huiqi Yuan [52], among others, found that ethanol in L02 cells increased the expression of Caspase-3, P53 and Ac-p53 in in vitro experiments. In the present study, TUNEL staining analysis of the tissues revealed that DHM was effective in restoring AFB1-induced apoptosis in hepatocytes and resulted in a significant reduction in apoptosis positivity. Notably, the expression levels of proteins of Caspase-3, Cleaved-caspase-3, Bax, and Bcl-2 were detected by Western blot. The results showed that the expression of apoptotic proteins Bax and Caspase-3 was up-regulated, while the expression of anti-apoptotic protein Bcl-2 was down-regulated in AFB1-treated liver tissues. However, the DHM administration reversed these trends. This indicates that DHM has anti-apoptotic activity and has a better mitigating effect on liver injury in mice. 

## 5. Conclusions

In conclusion, this study investigated the protective effect of DHM against AFB1-induced liver injury. In the first instance, based on network pharmacology and molecular docking techniques, DHM was found to have a significant ameliorative effect on AFT-induced liver injury, and it may exert its anti-hepatic injury effect by inhibiting the VEGF signalling pathway and suppressing the overexpression of Caspase3, HIF1A, SRC and VEGFA proteins. Interestingly, DHM administration was found to significantly alleviate AFB1-induced histological damage by animal experiments, and DHM was found to exert its antioxidant capacity by modulating the Nrf2/HO-1 antioxidant pathway. Importantly, DHM treatment resulted in the down-regulation of the expression of pro-apoptotic proteins Bax and Caspase-3 and the up-regulation of the expression of the anti-apoptotic protein Bcl-2. This indicates that DHM can reduce apoptosis in mouse liver tissue and has a better mitigating effect on liver injury in mice. Taken together, these results provide valuable guidance for further study of DHM in the treatment of liver-related diseases and lay the foundation for the in-depth development and utilization of DHM. However, the specific mechanism of this effect is still unclear, and further research is needed to evaluate the effect of DHM on AFB 1-induced liver injury. 

## Figures and Tables

**Table 1:** Binding energies of DHM to four target proteins, CASP3, HIF1A, SRC, and VEGFA.

Target Protein	Combined Heat and Power (kcal/mol)
CASP3	−5.03
HIF1A	−7.8
SRC	−6.4
VEGFA	−5.86

## Data Availability

All data in this article are presented in the article, and the original data are available from the corresponding author upon reasonable request.
